# Supplementary material for: Chromatographic Method for Monitoring of Pesticide Residues and Risk Assessment for Herbal Decoctions Used in Traditional Korean Medicine Clinics
Source: Molecules. 2023 Apr 10;28(8):3343. doi: 10.3390/molecules28083343 (PMC10142989; doi:10.3390/molecules28083343)
Supplement: Supplementary file 1 [file molecules-28-03343-s001.zip › Supplementary Materials File S1.pdf]

molecules

MDPI

Supplementary Materials File S1

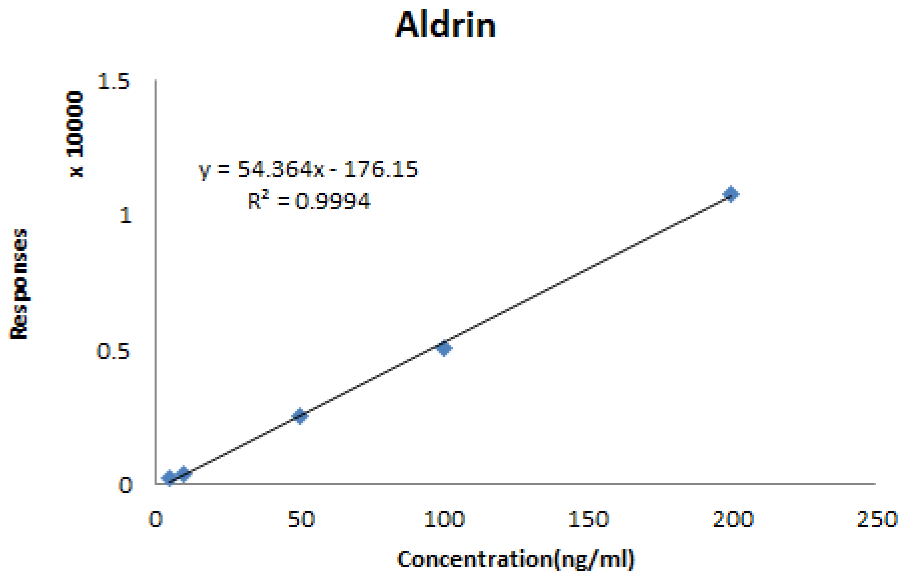

Figure S1. GC-MS/MS standard solution calibration curve (Aldrin)**.** GC: gas chromatography; MS: mass spectrometry.

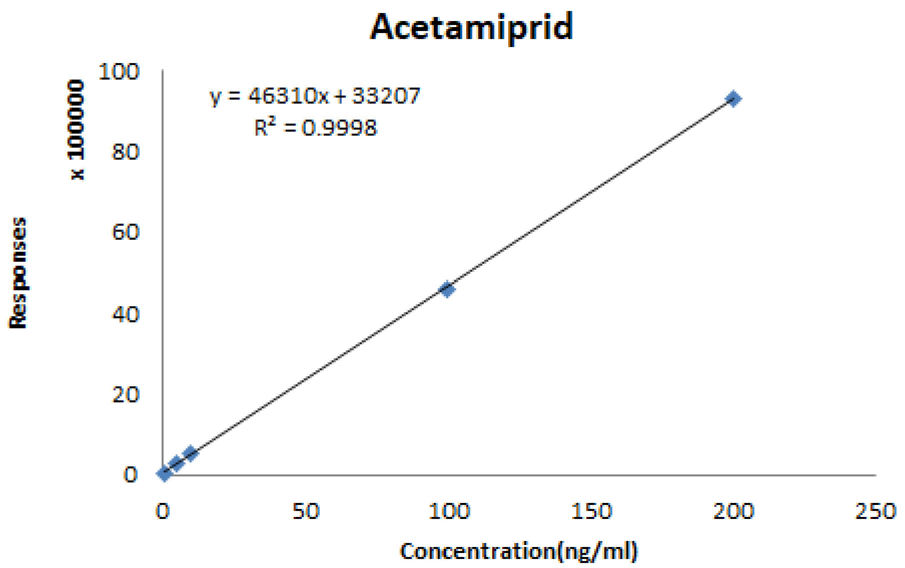

Figure S2. LC-MS/MS standard solution calibration curve (Acetamiprid); LC: liquid chromatography; MS: mass spectrometry.
