# Supplementary material for: Chromatographic Method for Monitoring of Pesticide Residues and Risk Assessment for Herbal Decoctions Used in Traditional Korean Medicine Clinics
Source: Molecules. 2023 Apr 10;28(8):3343. doi: 10.3390/molecules28083343 (PMC10142989; doi:10.3390/molecules28083343)
Supplement: Supplementary file 1 [file molecules-28-03343-s001.zip › Supplementary Materials File S2.pdf]

molecules

MDPI

Supplementary Materials File S2

Table S1. GC-MS/MS analysis pesticide standard solution slope and correlation (R^2).

NO.	Compound	Slope	y-Intercept	R^2	NO.	Compound	Slope	y-Intercept	R^2
1	Acrinathrin-1	2.70E+01	-4.37E+01	0.996	69	Fluopyram	7.38E+03	-5.06E+04	0.999
2	Acrinathrin-2	7.59E+01	-4.41E+02	0.995	70	Fonofos	4.18E+03	-1.62E+04	0.997
3	Alachlor	2.17E+03	-9.91E+03	0.996	71	Fthalide	1.46E+03	-7.43E+03	0.995
4	Aldrin	5.46E+01	-1.33E+02	0.999	72	Halfenprox	5.92E+02	-3.35E+03	0.990
5	Ametoctradin	3.42E+01	-3.31E+02	0.992	73	Heptachlor	3.66E+03	-6.41E+03	0.999
6	Anilofos	7.13E+02	-4.85E+03	0.983	74	Heptachlor-epoxide	9.14E+02	-2.11E+03	0.999
7	Azaconazole	8.73E+03	-4.24E+04	0.995	75	Imibenconazole	2.46E+02	-8.47E+02	0.997
8	Benfuresate	2.41E+03	-8.57E+03	0.996	76	Indanofan	2.43E+02	-1.52E+03	0.993
9	BHC(alpha)	2.76E+03	-4.47E+03	0.999	77	Indoxacarb	4.69E+01	-3.23E+02	0.991
10	BHC(beta)	2.09E+03	-3.55E+03	0.998	78	Iprodione	1.28E+02	-8.97E+02	0.993
11	BHC(delta)	1.77E+03	-6.85E+03	0.998	79	Isazofos	3.65E+03	-1.68E+04	0.996
12	BHC(gamma)	2.28E+03	-2.37E+03	0.998	80	Mecarbam	7.97E+02	-5.15E+03	0.990
13	Bifenox	2.57E+02	-1.84E+03	0.991	81	Methidation	4.59E+03	-2.82E+04	0.991
14	Bifenthrin	1.40E+04	-7.26E+04	0.993	82	Metolachlor	8.04E+03	-3.61E+04	0.996
15	Bromobutide	5.23E+02	-2.59E+03	0.996	83	Metribuzin	1.62E+03	-8.84E+03	0.994
16	Bromopropylate	2.39E+03	-1.24E+04	0.992	84	o,p-DDT	1.05E+04	-9.36E+03	0.996
17	Butachlor	1.08E+03	-4.91E+03	0.994	85	Oxyfluorfen	2.47E+02	-1.59E+03	0.991
18	Butafenacil	2.97E+03	-1.47E+04	0.992	86	p,p-DDD	1.08E+04	-9.57E+03	0.996
19	Carbophenothion	5.77E+02	-3.34E+03	0.991	87	P,P-DDE	7.54E+03	-2.26E+04	0.999
20	Chlorantraniliprole	1.03E+03	-9.82E+03	0.993	88	p,p-DDT	8.61E+03	-4.39E+04	0.995
21	Chlordane(cis)	1.15E+03	-2.54E+03	0.999	89	Parathion-ethyl	1.09E+03	-6.77E+03	0.989
22	Chlordane(trans)	7.04E+02	-1.71E+03	0.999	90	Parathion-methyl	1.48E+03	-1.01E+04	0.991
23	Chlorfenapyr	1.19E+02	-1.10E+03	0.994	91	Pendimethalin	7.24E+02	-4.10E+03	0.991
24	Chlorfenvinphos(cis)	1.76E+03	-1.04E+04	0.990	92	Penthiopyrad	1.28E+03	-6.08E+03	0.993
25	Chlorfenvinphos(trans)	1.77E+03	-1.03E+04	0.991	93	Permethrin-1	2.89E+02	-1.49E+03	0.994
26	Chlorfluazuron	2.00E+02	-1.10E+03	0.996	94	Permethrin-2	4.52E+02	-2.50E+03	0.992
27	Chlorobenzilate	5.12E+03	-2.51E+04	0.993	95	Phenothrin-1	3.95E+02	-2.24E+03	0.991
28	Chlorpropham	8.37E+02	-4.74E+03	0.995	96	Phenothrin-2	3.51E+02	-2.01E+03	0.991
29	Chlorpyrifos-methyl	2.97E+03	-1.42E+04	0.996	97	Phorate	2.79E+01	4.80E+01	0.995
30	Cyfluthrin	7.11E+01	-3.14E+02	0.996	98	Phorate oxon	7.43E+02	-2.96E+03	0.996
31	Cyhalothrin-1	7.83E+02	-4.32E+03	0.991	99	Phorate sulfone	1.83E+03	-1.09E+04	0.992
32	Cyhalothrin-2	1.35E+03	-7.91E+03	0.990	100	Phorate sulfoxide	4.70E+03	-2.70E+04	0.992
33	Cypermethrin	2.27E+02	-1.45E+03	0.992	101	Phosalone	1.74E+03	-1.10E+04	0.990
34	Cyprodinil	2.91E+03	-1.47E+04	0.995	102	Picoxystrobin	1.21E+03	-6.90E+03	0.993
35	Deltamethrin	4.73E+02	-2.62E+03	0.993	103	Piperonyl butoxide	2.15E+03	-1.22E+04	0.993
36	Diclofop-methyl	1.46E+03	-7.58E+03	0.993	104	Pirimiphos-ethyl	6.06E+02	-3.24E+03	0.995
37	Dicloran (Botran)	3.31E+02	-2.39E+03	0.997	105	Pretilachlor	3.37E+03	-1.66E+04	0.995
38	Dicofol	3.21E+03	-1.65E+04	0.994	106	Prochlora	7.39E+01	-5.41E+02	0.991
39	Dieldrin	6.05E+02	-8.43E+02	0.999	107	Procymidone	1.38E+03	-5.96E+03	0.997
40	Difenoconazole-1	1.30E+03	-6.74E+03	0.993	108	Promecarb	2.12E+03	-9.56E+03	0.996
41	Difenoconazole-2	1.03E+03	-4.14E+03	0.983	109	Prometryn	1.81E+03	-9.43E+03	0.996
42	Dimethoate	3.29E+02	-2.39E+03	0.990	110	Propachlor	1.61E+03	3.34E+05	0.998
43	Dimethylvinphos	1.83E+03	-1.14E+04	0.992	111	Propazine	2.45E+03	-1.11E+04	0.997
44	Dimethylvinphos-1	1.83E+03	-1.23E+04	0.989	112	Propiconazole-1	9.37E+02	-4.59E+03	0.991
45	Diphenylamine	4.10E+03	-2.20E+04	0.996	113	Propiconazole-2	5.29E+02	-1.58E+03	0.993
46	Disulfoton	7.58E+02	-2.92E+03	0.997	114	Propisochlor	8.97E+02	-4.63E+03	0.996

47	Endosulfan(alpha)	3.30E+02	-5.76E+02	0.999	115	Propyzamide	6.39E+03	-3.98E+04	0.994
48	Endosulfan(beta)	3.27E+02	-1.00E+03	0.998	116	Prothiofos	2.12E+03	-1.02E+04	0.995
49	Endosulfan-sulfate	2.34E+02	-1.07E+03	0.996	117	Quintozene	9.98E+02	-3.38E+03	0.997
50	Endrin	7.96E+02	-2.49E+03	0.998	118	Silafluofen	4.53E+03	-2.39E+04	0.992
51	EPN	9.33E+02	-4.79E+03	0.992	119	Simazine	1.97E+02	-1.37E+03	0.993
52	Epoxiconazole	4.65E+03	-2.49E+04	0.991	120	Simeconazole	5.45E+03	-2.84E+04	0.994
53	Ethalfluralin	7.63E+02	-2.00E+03	0.994	121	Simetryn	4.21E+02	-3.01E+03	0.991
54	Ethion	2.91E+03	-1.69E+04	0.992	122	sofenphos	4.98E+03	-2.63E+04	0.994
55	Etridiazole	2.30E+03	-3.70E+03	0.991	123	Spiromesifen	3.93E+02	-2.35E+03	0.993
56	Fenclorim	6.02E+03	-1.43E+04	0.998	124	Tebupirimfos	2.91E+03	-1.05E+04	0.997
57	Fenitrothion	7.00E+02	-4.58E+03	0.995	125	Tefluthrin	8.91E+03	-3.27E+04	0.998
58	Fenothiocarb	1.59E+03	-1.06E+04	0.986	126	Terbufos	4.42E+03	-1.73E+04	0.997
59	Fenoxanil	1.30E+02	-9.13E+02	0.993	127	Terbutryn	1.85E+03	-9.10E+03	0.995
60	Fenpropathrin (Danitol)	6.68E+02	-3.95E+03	0.991	128	Tetradifon	3.45E+03	-1.45E+04	0.997
61	Fenthion	2.99E+03	-1.59E+04	0.994	129	Thifluzamide	2.10E+03	-9.61E+03	0.993
62	Fenvalerate-1	9.64E+01	-5.81E+02	0.993	130	Tolclofos-methyl	4.59E+03	-1.79E+04	0.997
63	Fenvalerate-2	1.69E+02	-9.83E+02	0.983	131	Triadimenol	1.26E+03	-5.99E+03	0.993
64	Fipronil	8.18E+02	-4.65E+03	0.992	132	Tri-allate	2.80E+03	-8.28E+03	0.997
65	Fipronil sulfone	4.56E+01	-2.75E+02	0.999	133	Trifluralin	5.68E+02	-2.13E+03	0.995
66	Flucythrinate-1	2.35E+02	-1.45E+03	0.991	134	Vinclozolin	1.05E+03	-4.65E+03	0.995
67	Flucythrinate-2	3.83E+02	-1.76E+03	0.995	135	Zoxamide	3.94E+02	-1.92E+03	0.999
68	Flumioxazine	8.81E+01	-5.43E+02	0.991					

GC: gas chromatography; MS: mass spectrometry.

Table S2. LC-MS/MS analysis pesticide standard solution slope and correlation (R^2).

NO.	Compound	Slope	y-Intercept	R^2	NO.	Compound	Slope	y-Intercept	R^2
1	Abamectin B1	1.07E+03	-2.18E+03	0.998	105	Isopyrazam	3.82E+04	3.88E+04	0.999
2	Acephate	6.50E+03	-7.37E+03	0.999	106	Kresoxim-methyl	1.20E+04	1.51E+04	0.997
3	Acetamiprid	4.63E+04	3.32E+04	1.000	107	Linuron	9.69E+03	1.52E+04	0.996
4	Aldicarb	1.95E+04	2.02E+04	0.994	108	Lufenuron(-)	2.08E+03	1.80E+04	0.987
5	Amisulbrom	8.48E+02	8.23E+01	1.000	109	Malathion	9.34E+03	1.26E+03	1.000
6	Azimsulfuron	1.35E+04	-1.61E+04	1.000	110	Mandipropamid	3.71E+04	-1.34E+04	1.000
7	Azinphos-methyl	3.66E+01	7.86E+01	0.977	111	Mefenacet	8.44E+04	-1.18E+05	0.997
8	Azoxystrobin	9.26E+04	9.65E+04	0.999	112	Mepanipyrim	1.25E+04	-8.77E+03	1.000
9	Bendiocarb	1.78E+04	2.92E+04	0.999	113	Mepronil	7.04E+04	2.43E+05	0.992
10	Bensulfuron_methyl	2.14E+04	-3.37E+04	1.000	114	Metalaxyl	7.66E+04	3.97E+04	1.000
11	Benthiavalicarb_iso-propyl	2.85E+04	-1.41E+04	1.000	115	Metamifop	5.72E+04	6.10E+04	0.998
12	Benzobicyclon	1.36E+04	4.18E+02	1.000	116	Metazosulfuron	3.18E+03	-8.02E+02	1.000
13	Benzoximate	4.35E+04	8.17E+03	1.000	117	Metconazole	1.25E+04	5.57E+03	0.998
14	Bitertanol	4.16E+03	-1.57E+04	0.997	118	Methabenzthiazuron	1.79E+04	1.54E+04	0.998
15	Boscalid	1.34E+04	1.01E+04	0.999	119	Methiocarb	3.09E+04	7.38E+03	0.998
16	Bromacil	1.27E+00	5.18E+03	0.915	120	Methomyl	1.34E+04	-4.43E+03	1.000
17	Buprofezin	1.28E+05	8.19E+04	0.999	121	Methoxyfenozide	3.61E+04	9.45E+04	0.996
18	Cadusafos	6.20E+04	6.77E+02	1.000	122	Metobromuron	5.62E+03	-9.94E+03	0.999
19	Cafenstrole	7.71E+03	-3.67E+03	0.999	123	Metolcarb	1.77E+04	-6.45E+04	0.981
20	Carbaryl	9.92E+03	1.44E+04	0.998	124	Metrafenone	4.52E+04	8.53E+03	1.000
21	Carbendazim	3.21E+03	-3.47E+03	0.999	125	Mevinphos	7.80E+03	1.98E+04	0.993
22	Carbofuran	5.54E+04	7.37E+04	1.000	126	Milbemectin A3	5.62E+02	2.99E+03	1.000
23	Carboxin	5.77E+04	6.68E+04	0.998	127	Milbemectin A4	1.23E+03	-1.42E+03	0.999
24	Carfentrazone_ethyl	7.17E+03	-2.38E+03	1.000	128	Molinate	6.43E+03	-1.38E+04	0.996

25	Carpropamide	2.70E+04	2.44E+03	1.000	129	Monocrotophos	2.31E+04	4.25E+03	1.000
26	Chlorpyrifos	1.12E+04	-1.26E+03	1.000	130	Myclobutanil	8.56E+03	-1.94E+04	0.995
27	Chlorsulfuron	3.95E+03	-6.82E+03	0.999	131	Napropamide	4.13E+04	-4.58E+04	1.000
28	Chromafenozide	6.22E+04	-9.76E+04	0.999	132	Nicosulfuron	1.48E+04	3.87E+04	0.993
29	Clofentezine	7.14E+03	7.89E+03	0.999	133	Novaluron	3.94E+03	2.21E+03	0.999
30	Clomazone	5.80E+04	1.02E+04	1.000	134	Nuarimol	3.93E+03	-1.88E+03	1.000
31	Clothianidin	1.03E+04	8.03E+03	1.000	135	Ofurace	2.36E+04	3.95E+04	0.999
32	Cyazofamid	1.31E+04	-8.75E+03	1.000	136	Omethoate	8.97E+03	5.65E+04	0.958
33	Cyclosulfamuron	2.93E+04	-9.57E+04	0.995	137	Oxadiazon	9.65E+03	-9.26E+03	1.000
34	Cyflufenamid	2.69E+04	2.39E+04	1.000	138	Oxadixyl	3.18E+04	1.70E+05	0.968
35	Cyhalofop-butyl	6.74E+01	8.40E+02	0.934	139	Oxamyl	1.45E+04	-1.85E+04	1.000
36	Cymoxanil	3.89E+00	6.77E+02	0.979	140	Oxaziclomefone	9.90E+04	-2.45E+04	1.000
37	Cyproconazole(I_II)	6.50E+03	1.11E+03	1.000	141	Paclobutrazol	1.36E+04	2.59E+04	0.993
38	Daimuron	8.00E+04	8.56E+04	1.000	142	Penconazole	1.37E+04	1.10E+04	0.999
39	Demeton-S-methyl	7.77E+02	1.46E+03	0.994	143	Pencycuron	1.12E+05	-5.94E+04	1.000
40	Diazinon	6.42E+04	-1.87E+04	1.000	144	Penoxsulam	2.49E+03	1.15E+03	0.998
41	Dichlorvos(DDVP)	5.15E+03	6.08E+03	1.000	145	Pentoxazone	4.85E+01	1.55E+02	0.999
42	Diethofencarb	2.52E+04	8.37E+04	0.990	146	Phenthoate	1.89E+04	2.98E+04	0.996
43	Diflubenzuron	1.18E+04	-5.11E+03	1.000	147	Phosphamidon	8.47E+03	2.41E+04	0.994
44	Dimepiperate	4.52E+04	-2.84E+04	1.000	148	Phoxim	8.54E+03	-1.67E+03	1.000
45	Dimethametryn	1.23E+05	-7.32E+04	1.000	149	Piperophos	4.76E+04	4.04E+04	0.999
46	Dimethenamid	1.00E+05	-9.14E+03	0.999	150	Pirimicarb	4.75E+04	8.33E+04	1.000
47	Dimethomorph(E,Z)	3.04E+04	-6.68E+04	0.999	151	Pirimiphos-methyl	3.49E+04	-1.09E+04	1.000
48	Diniconazole	5.23E+03	-4.18E+03	1.000	152	Probenazole	1.24E+03	9.02E+03	0.993
49	Dinotefuran	1.32E+04	-7.69E+03	1.000	153	Profenofos	2.55E+04	2.74E+04	0.998
50	Diphenamid	1.12E+05	-7.01E+04	1.000	154	Propamocarb	5.61E+04	-1.21E+05	0.998
51	Dithiopyr	1.05E+04	-5.81E+03	1.000	155	Propanil	2.75E+03	1.06E+04	0.995
52	Diuron	8.27E+03	-1.32E+04	1.000	156	Propaquizafop	1.66E+04	1.10E+04	0.999
53	Edifenphos	5.14E+04	4.98E+04	0.998	157	Propoxur	1.92E+04	1.08E+05	0.971
54	Esprocarb	6.00E+04	7.94E+04	0.998	158	Pyraclofos	2.66E+04	-5.78E+02	1.000
55	Ethaboxam	5.59E+03	1.69E+04	0.997	159	Pyraclostrobin	3.91E+04	3.48E+04	0.999
56	Ethiofencarb	2.76E+03	5.49E+03	0.998	160	Pyrazolate	2.05E+04	3.50E+04	0.997
57	Ethofenprox	8.77E+04	-4.67E+03	1.000	161	Pyrazophos	2.19E+04	-4.66E+04	0.999
58	Ethoprophos	3.15E+04	-9.88E+04	0.996	162	Pyribenzoxim	3.39E+04	1.55E+04	0.999
59	Ethoxysulfuron	1.72E+04	4.56E+03	0.999	163	Pyributicarb	8.72E+04	7.09E+04	1.000
60	Etoxazole	9.81E+04	7.12E+04	0.999	164	Pyridaben	8.77E+04	-2.57E+04	1.000
61	Etrimfos	3.86E+04	7.51E+03	1.000	165	Pyridaphenthion	3.62E+04	-1.03E+05	0.992
62	Famoxadone	1.12E+04	-1.62E+04	1.000	166	Pyrifluquinazon	1.51E+04	-2.32E+04	0.999
63	Fenamiphos	6.21E+04	-3.47E+03	1.000	167	Pyriftalid	1.08E+04	1.16E+04	1.000
64	Fenarimol	1.49E+03	1.63E+03	1.000	168	Pyrimethanil	9.13E+03	-2.87E+03	1.000
65	Fenazaquin	5.33E+04	-4.09E+04	1.000	169	Pyrimidifen	1.77E+05	2.07E+04	1.000
66	Fenbuconazole	7.86E+03	-3.65E+02	1.000	170	Pyriminobac-methyl(E)	4.80E+04	-2.21E+04	1.000
67	Fenhexamid	5.05E+03	-3.40E+03	0.999	171	Pyriminobac-methyl(Z)	9.45E+04	2.41E+05	0.992
68	Fenobucarb	2.62E+04	1.77E+04	0.998	172	Pyrimisulfan	2.57E+04	3.21E+04	0.996
69	Fenoxaprop-ethyl	7.33E+04	1.08E+05	0.998	173	Pyriproxyfen	5.61E+04	1.27E+04	1.000
70	Fenoxycarb	2.01E+04	2.32E+04	0.997	174	Pyroquilon	3.27E+04	-4.57E+04	0.999
71	Fenpyroximate	7.57E+04	-5.44E+04	1.000	175	Quinalphos	1.89E+04	2.02E+04	0.999
72	Fentrazamide	3.98E+03	5.59E+03	0.999	176	Quinmerac	2.78E+04	1.15E+05	0.991
73	Ferimzone(E,Z)	7.21E+04	3.34E+04	0.999	177	Quinoclamine	2.69E+03	1.53E+04	0.991

74	Flonicamid	4.82E+03	-4.00E+03	1.000	178	Quizalofop-ethyl	7.99E+04	1.09E+04	1.000
75	Fluacrypyrim	9.39E+04	4.90E+04	0.999	179	Saflufenacil	1.94E+03	-3.12E+03	0.998
76	Flubendiamide	1.49E+03	-3.91E+03	0.999	180	Sethoxydime	7.17E+03	3.85E+02	0.991
77	Flucetosulfuron	6.66E+03	-1.01E+04	1.000	181	Spinetoram_J	3.53E+04	-5.51E+04	0.999
78	Fludioxonil	2.90E+03	2.23E+03	1.000	182	Spinetoram_L	9.93E+03	-6.21E+03	1.000
79	Flufenacet	5.77E+04	-4.44E+04	1.000	183	Spirodiclofen	1.39E+04	-1.02E+04	1.000
80	Flufenoxuron	1.21E+04	-1.36E+04	1.000	184	Spirotetramat	1.86E+04	-1.53E+04	1.000
81	Fluopicolide	2.21E+04	3.50E+04	0.998	185	Sulfoxaflor	6.49E+03	1.45E+04	0.997
82	Fluquinconazole	3.11E+03	-5.10E+03	0.999	186	Tebuconazole	1.19E+04	-8.57E+03	1.000
83	Flusilazole	3.82E+04	1.17E+03	1.000	187	Tebufenozide	4.21E+04	2.07E+04	0.999
84	Flutolanil	6.10E+04	8.04E+04	0.999	188	Tebufenpyrad	1.51E+04	1.84E+04	0.999
85	Fluxapyroxad	2.72E+04	1.50E+05	0.983	189	Teflubenzuron(-)	3.94E+03	1.86E+04	0.994
86	Forchlorfenuron	1.42E+04	4.29E+04	0.991	190	Terbuthylazine	6.09E+04	1.34E+05	0.997
87	Fosthiazate	3.88E+04	7.93E+04	0.995	191	Tetraconazole	9.56E+03	-1.84E+04	0.997
88	Furathiocarb	5.71E+04	7.47E+03	1.000	192	Thenylchlor	4.74E+04	-1.20E+05	0.999
89	Gibberellic acid	1.24E+02	2.89E+02	0.997	193	Thiabendazole	6.06E+04	1.45E+05	0.997
90	Halosulfuron-methyl	1.26E+04	-6.09E+04	0.985	194	Thiacloprid	4.43E+04	1.01E+05	1.000
91	Haloxyfop	3.48E+03	-5.03E+03	0.999	195	Thiamethoxam	3.17E+04	2.02E+04	0.999
92	Hexaconazole	9.22E+03	-4.20E+03	1.000	196	Thiazopyr	1.39E+04	2.34E+04	0.996
93	Hexaflumuron(-)	7.42E+03	2.47E+04	0.998	197	Thidiazuron	7.13E+02	5.14E+03	0.999
94	Hexazinone	7.41E+04	7.96E+04	1.000	198	Thifensulfuron-me-thyl	3.47E+02	-2.40E+02	0.993
95	Hexythiazox	3.72E+04	-3.29E+04	1.000	199	Thiobencarb	5.94E+04	1.80E+04	1.000
96	Imazalil	1.08E+04	-2.81E+03	1.000	200	Thiodicarb	8.22E+03	-3.69E+03	1.000
97	Imazosulfuron	3.46E+03	-2.32E+03	1.000	201	Tiadinil(-)	1.64E+03	-4.53E+02	1.000
98	Imicyafos	5.56E+04	9.99E+04	1.000	202	Triadimefon	8.91E+03	-1.29E+04	0.998
99	Imidacloprid	1.38E+04	-1.22E+04	1.000	203	Triazophos	5.34E+04	-2.69E+04	0.999
100	Inabenfide	9.00E+03	-9.84E+03	1.000	204	Tricyclazole	4.36E+04	-1.56E+04	1.000
101	Iprobenfos	3.70E+04	5.09E+04	0.995	205	Trifloxystrobin	9.81E+04	7.10E+04	0.999
102	Iprovalicarb	5.17E+04	-5.45E+04	1.000	206	Triflumizole	9.42E+04	-1.45E+04	1.000
103	Isoprocarb	1.77E+04	-7.83E+03	1.000	207	Triflumuron	1.32E+04	9.98E+03	0.999
104	Isoprothiolane	7.50E+04	6.00E+04	0.999					

LC: liquid chromatography; MS: mass spectrometry.

Table S3. Average recovery and RSD of 113 pesticides spiked in herbal decoctions at three different concentrations with GC-MS/MS(n=3).

No.	Pesticides	LOD(ng/g)	LOQ(ng/g)	Recovery,% (RSD,%)		
				10 ng/g	50 ng/g	100 ng/g
1	Acrinathrin	2.0	6.6	98(1)	91(3)	99(1)
2	Alachlor	1.6	5.4	100(3)	99(1)	106(1)
3	Aldrin	3.0	9.0	119(3)	107(2)	109(1)
4	Ametoctradin	2.3	7.5	108(8)	97(2)	100(2)
5	Anilofos	0.2	0.8	98(1)	98(2)	107(1)
6	Azaconazole	0.4	1.0	100(9)	100(1)	105(1)
7	Benfurasate	1.6	5.4	103(4)	101(1)	105(1)
8-1	BHC(alpha)	2.4	7.8	102(1)	101(1)	107(1)
8-2	BHC(beta)	1.4	4.4	99(2)	99(1)	103(2)
8-3	BHC(delta)	1.8	6.0	105(1)	101(1)	106(1)
8-4	BHC(gamma)	1.8	6.0	100(1)	102(1)	108(1)
9	Bifenox	3.8	12.4	101(2)	86(3)	99(2)
10	Bifenthrin	0.4	1.2	101(1)	98(1)	103(1)

11	Bromobutide	1.2	4.4	103(5)	98(2)	103(1)
12	Bromopropylate	0.8	2.8	101(1)	97(1)	103(1)
13	Butachlor	1.2	4.4	97(7)	99(1)	103(1)
14	Butafenacil	1.4	4.4	99(1)	92(1)	100(1)
15	Carbophenothion	2.0	6.6	100(1)	94(1)	102(1)
16	Chlorantraniliprole	1.4	5.0	100(3)	101(2)	101(1)
17-1	Chlordane-cis	0.4	1.2	102(2)	100(1)	106(1)
17-2	Chlordane-trans	3.0	9.0	102(2)	100(1)	106(1)
18	Chlorfenapyr	2.4	8.2	104(3)	100(1)	105(1)
19	Chlorfenvinphos	1.2	4.4	1021)	99(2)	104(1)
20	Chlorfluazuron	1.0	3.2	102(8)	98(1)	104(1)
21	Chlorobenzilate	0.4	1.6	101(1)	100(1)	105(1)
22	Chlorpropham	1.0	3.0	98(3)	100(1)	105(1)
23	Chlorpyrifos-methyl	0.6	2.0	104(1)	101(2)	108(2)
24	Cyfluthrin	2.0	6.6	112(4)	96(3)	105(1)
25	Cyhalothrin	2.0	6.8	104(3)	92(1)	101(1)
26	Cypermethrin	1.8	6.0	104(2)	93(1)	102(2)
27	Cyprodinil	1.6	5.0	99(3)	100(1)	106(1)
28	Deltamethrin	3.1	9.2	97(1)	92(2)	99(1)
29	Diclofop-methyl	2.0	6.6	100(1)	100(1)	106(1)
30	Dicloran	0.6	2.0	97(5)	93(2)	101(1)
31	Dicofol	1.2	3.8	97(5)	99(2)	106(1)
32	Dieldrin	2.8	9.0	110(3)	101(2)	106(4)
33	Difenoconazole	2.4	8.2	101(14)	98(3)	106(1)
34	Dimethoate	2.6	8.6	99(6)	101(3)	107(1)
35	Dimethylvinphos	0.8	2.6	101(1)	101(1)	107(1)
36	Diphenylamine	1.0	3.2	103(1)	99(4)	105(1)
37	Disulfoton	3.0	9.8	102(1)	99(1)	105(1)
38-1	Endosulfan-alpha	2.8	9.6	103(2)	101(1)	105(2)
38-2	Endosulfan-beta	2.6	8.8	102(7)	103(1)	109(1)
38-3	Endosulfan sulfate	2.0	6.6	100(2)	101(1)	108(1)
39	Endrin	2.2	6.5	108(8)	95(9)	98(3)
40	EPN	0.6	2.4	99(4)	90(5)	97(1)
41	Epoxiconazole	1.8	6.0	104(7)	95(2)	102(1)
42	Ethalfluralin	2.2	7.6	100(1)	93(1)	100(1)
43	Ethion	0.6	2.2	110(5)	104(4)	99(3)
44	Etridiazole	4.1	12.3	105(5)	101(1)	105(1)
45	Fenclorim	2.8	9.4	102(1)	101(1)	107(1)
46	Fenitrothion	0.2	0.6	101(4)	92(2)	101(2)
47	Fenothiocarb	0.2	0.6	94(8)	95(1)	103(2)
48	Fenoxanil	0.4	1.4	103(1)	98(1)	104(1)
49	Fenpropathrin	0.8	2.8	90(5)	99(2)	101(2)
50	Fenthion	1.6	5.2	102(1)	102(2)	106(1)
51	Fenvalerate	0.8	2.6	98(3)	91(1)	99(1)
52	Fipronil	1.2	4.2	99(1)	93(1)	100(1)
53	Flucythrinat	1.2	4.2	98(1)	90(1)	99(1)
54	Flumioxazin	0.4	1.4	100(2)	90(2)	98(3)
55	Fluopyram	1.0	3.0	99(3)	97(1)	104(1)
56	Fonofos	1.6	5.4	101(2)	99(1)	105(1)
57	Fthalide	1.0	3.6	102(3)	100(1)	107(2)
58	Halfenprox	1.2	3.8	97(3)	88(1)	99(1)

59	Heptachlor	4.0	13.0	100(2)	97(1)	103(1)
60	Heptachlor-epoxide	2.4	8.2	102(2)	100(1)	106(1)
61	Imibenconazole	2.4	7.6	100(10)	109(8)	101(5)
62	Indanofan	7.0	20	1349(6)	241(32)	189(20)
63	Indoxacarb	1.4	4.4	93(9)	98(3)	105(4)
64	Iprodione	3.0	9.0	101(6)	101(2)	109(2)
65	Isazofos	1.2	4.0	110(3)	101(1)	106(3)
66	Isofenphos	1.2	4.2	102(1)	98(1)	104(1)
67	Mecarbam	0.4	1.0	97(3)	95(1)	103(1)
68	Methidathion	1.0	3.2	98(4)	101(1)	109(1)
69	Metolachlor	1.4	4.4	102(0)	101(1)	106(1)
70	Metribuzin	0.6	1.6	102(1)	97(2)	103(1)
71	Oxyfluorfen	3.4	11.4	102(2)	103(1)	105(1)
72-1	o,p-DDT	0.6	2.0	91(4)	90(3)	99(1)
72-2	p,p-DDD	3.0	9.0	101(1)	79(3)	101(3)
72-3	p,p'-DDE	2.2	7.4	97(9)	99(1)	106(1)
72-4	p,p-DDT	2.2	7.0	97(1)	89(7)	99(5)
73	Parathion-ethyl	0.4	1.4	101(1)	90(1)	98(1)
74	Parathion-methyl	0.4	1.2	101(1)	94(1)	102(1)
75	Pendimethalin	1.4	4.6	102(5)	89(3)	97(1)
76	Penthiopyrad	1.2	4.0	102(4)	96(1)	101(1)
77	Permethrin	3.0	9.0	101(1)	97(1)	102(1)
78	Phenothrin	0.6	2.0	105(7)	96(1)	102(1)
79	Phorate	1.8	5.6	103(1)	99(1)	104(1)
80	Phosalone	1.0	3.6	101(1)	98(1)	106(1)
81	Picoxystrobin	0.8	2.8	103(0)	97(1)	104(1)
82	Piperonyl butoxide	3.4	12	99(2)	95(2)	102(1)
83	Pirimiphos-ethyl	0.6	1.8	102(1)	98(1)	104(1)
84	Pretilachlor	1.0	3.6	102(1)	99(1)	105(1)
85	Prochloraz	0.2	0.8	139(7)	94(8)	117(6)
86	Procymidone	2.8	9.4	98(7)	1001()	106(1)
87	Promecarb	1.0	3.6	101(19)	97(6)	105(1)
88	Prometryn	0.2	0.8	100(5)	99(1)	105(1)
89	Propachlor	3.0	9.0	109(1)	102(3)	107(1)
90	Propazine	3.0	9.0	100(2)	101(1)	106(1)
91	Propiconazole	3.0	9.0	102(6)	96(1)	104(1)
92	Propisochlor	2.0	6.6	106(7)	102(1)	107(1)
93	Propyzamide	1.4	4.4	96(3)	98(1)	104(1)
94	Prothiofos	3.0	9.0	104(1)	98(1)	104(1)
95	Pyridalyl	3.0	9.0	100(3)	93(1)	101(1)
96	Quintozene	0.8	3.0	102(2)	96(1)	103(1)
97	Silafluofen	1.0	3.6	101(4)	96(1)	102(2)
98	Simazine	0.2	0.6	94(8)	101(2)	109(1)
99	Simeconazole	1.2	3.8	100(3)	100(1)	104(3)
100	Simetryn	0.6	1.8	103(3)	98(1)	105(1)
101	Spiromesifen	1.4	5.0	98(1)	100(2)	107(1)
102	Tebupirimfos	1.6	5.2	101(1)	100(1)	105(1)
103	Tefluthrin	1.6	5.6	102(1)	102(1)	106(1)
104	Terbufos	0.8	3.0	102(1)	99(1)	105(1)
105	Terbutryn	1.8	6.0	100(3)	99(1)	105(1)
106	Tetradifon	2.2	7.4	100(2)	99(1)	105(1)

107	Thifluzamide	2.0	6.8	101(3)	96(2)	104(1)
108	Tolclofos-methyl	1.8	6.2	106(1)	102(1)	106(1)
109	Triadimenol	0.8	2.8	105(6)	99(2)	104(1)
110	Tri-allate	1.8	6.0	102(1)	101(1)	106(1)
111	Trifluralin	1.2	4.2	100(1)	91(1)	98(1)
112	Vinclozolin	0.4	1.6	98(3)	99(1)	105(1)
113	Zoxamide	0.4	1.4	95(2)	103(4)	113(5)

LOD: limit of detection, LOQ: limit of quantification, RSD: relative standard deviation.

Table S4. Average recovery and RSD of 207 pesticides spiked in herbal decoctions at three different concentrations with LC-MS/MS(n=3).

No.	Pesticides	LOD(ng/g)	LOQ(ng/g)	Recovery,% (RSD,%)		
				10 ng/g	50 ng/g	100 ng/g
1	Abamectin B1	0.39	1.21	99(10)	105(8)	107(7)
2	Acephate	0.39	1.21	88(4)	109(3)	81(1)
3	Acetamiprid	0.05	0.16	93(4)	107(5)	99(2)
4	Aldicarb	0.39	1.21	83(10)	103(8)	117(3)
5	Amisulbrom	0.39	1.21	111(5)	116(8)	119(3)
6	Azimsulfuron	0.05	0.16	110(2)	103(4)	111(6)
7	Azinphos-methyl	0.05	0.16	105(1)	83(5)	89(3)
8	Azoxystrobin	0.05	0.16	116(1)	94(6)	117(9)
9	Bendiocarb	0.05	0.16	79(9)	109(6)	111(3)
10	Bensulfuron-methyl	0.05	0.16	75(5)	84(5)	117(5)
11	Benthiavalicarb-isopropyl	0.39	1.21	77(2)	118(4)	100(3)
12	Benzobicyclon	0.39	1.21	93(14)	115(6)	118(3)
13	Benzoximate	0.39	1.21	117(1)	112(5)	117(2)
14	Bitertanol	0.39	1.21	97(9)	104(3)	100(4)
15	Boscalid	0.39	1.21	88(11)	88(8)	112(8)
16	Bromacil	0.39	1.21	103(5)	103(3)	107(2)
17	Buprofezin	0.05	0.16	106(5)	110(4)	116(2)
18	Cadusafos	0.05	0.16	105(3)	110(3)	107(4)
19	Cafenstrole	0.39	1.21	119(8)	108(8)	117(6)
20	Carbaryl	0.05	0.16	118(3)	116(5)	112(2)
21	Carbendazim	0.01	0.04	90(2)	106(9)	93(7)
22	Carbofuran	0.13	0.40	110(12)	102(7)	106(4)
23	Carboxin	0.13	0.40	117(5)	113(7)	110(6)
24	Carfentrazone-ethyl	0.39	1.21	113(7)	98(11)	112(4)
25	Carpropamide	0.39	1.21	71(5)	111(5)	115(3)
26	Chlorpyrifos	0.05	0.16	111(1)	105(2)	103(3)
27	Chlorsulfuron	0.13	0.40	106(10)	106(9)	107(7)
28	Chromafenozide	0.39	1.21	93(3)	115(9)	116(9)
29	Clethodim	0.39	1.21	91(7)	108(4)	106(6)
30	Clofentezine	0.39	1.21	96(6)	105(2)	98(4)
31	Clomazone	0.13	0.40	114(6)	114(1)	106(3)
32	Clothianidin	0.13	0.40	103(8)	113(2)	99(2)
33	Cyazofamid	0.39	1.21	65(6)	104(2)	119(3)
34	Cyclosulfamuron	0.39	1.21	79(10)	107(6)	104(4)
35	Cyflufenamid	0.39	1.21	161(10)	149(6)	112(3)
36	Cyhalofop-butyl	0.39	1.21	102(7)	100(5)	103(8)
37	Cymoxanil	0.39	1.21	87(8)	105(3)	104(3)
38	Cyproconazole(I, II)	0.39	1.21	75(8)	90(3)	113(3)

39	Dichlorvos(DDVP)	0.26	0.81	78(9)	112(7)	111(8)
40	Demeton-S-Methyl	0.39	1.21	101(9)	105(9)	106(7)
41	Diazinon	0.26	0.81	96(2)	110(3)	108(2)
42	Diethofencarb	0.05	0.16	77(7)	118(4)	103(4)
43	Diflubenzuron	0.26	0.81	102(7)	76(4)	113(3)
44	Dimepiperate	0.39	1.21	107(4)	104(2)	105(4)
45	Dimethametryn	0.13	0.40	87(13)	100(9)	103(5)
46	Dimethenamid	0.39	1.21	74(9)	100(5)	111(2)
47	Dimethomorph(E, Z)	0.39	1.21	85(10)	94(4)	108(2)
48	Diniconazole	0.39	1.21	99(8)	109(10)	109(6)
49	Dinotefuran	0.26	0.81	71(8)	98(2)	104(5)
50	Diphenamid	0.39	1.21	108(5)	114(5)	109(6)
51	Dithiopyr	0.39	1.21	118(11)	111(8)	108(8)
52	Diuron	0.13	0.40	95(10)	91(5)	108(6)
53	Daimuron	0.39	1.21	101(4)	105(5)	100(3)
54	Edifenphos	0.39	1.21	107(4)	112(1)	114(3)
55	Esprocarb	0.26	0.81	95(9)	101(6)	116(8)
56	Ethaboxam	0.39	1.21	85(9)	114(10)	117(2)
57	Ethiofencarb	0.05	0.16	87(12)	102(11)	111(3)
58	Etofenprox	0.26	0.81	88(2)	106(5)	116(3)
59	Ethoprophos	0.39	1.21	92(2)	109(8)	108(4)
60	Ethoxysulfuron	0.39	1.21	112(11)	80(6)	110(5)
61	Etoxazole	0.39	1.21	117(11)	113(5)	104(4)
62	Etrimfos	0.05	0.16	92(4)	107(9)	109(6)
63	Famoxadone	0.39	1.21	87(11)	110(8)	111(3)
64	Fenamiphos	0.39	1.21	93(6)	91(10)	105(3)
65	Fenarimol	0.05	0.16	92(12)	94(4)	112(2)
66	Fenazaquin	0.39	1.21	117(7)	91(4)	116(1)
67	Fenbuconazole	0.39	1.21	79(7)	84(11)	111(5)
68	Fenhexamid	0.39	1.21	83(3)	119(7)	112(4)
69	Fenobucarb	0.05	0.16	71(5)	104(5)	105(4)
70	Fenoxaprop-ethyl	0.39	1.21	140(13)	123(7)	110(2)
71	Fenoxycarb	0.39	1.21	91(11)	73(4)	110(4)
72	Fenpyroximate	0.13	0.40	106(3)	106(4)	100(3)
73	Fentrazamide	0.39	1.21	114(7)	110(10)	110(3)
74	Ferimzone(E,Z)	0.39	1.21	98(4)	91(3)	109(4)
75	Fipronil sulfone	0.39	1.21	109(13)	103(5)	100(7)
76	Flonicamid	0.05	0.16	99(3)	110(6)	94(7)
77	Fluacrypyrim	0.39	1.21	101(6)	115(12)	110(8)
78	Flubendiamide	0.39	1.21	104(8)	115(11)	100(9)
79	Flucetosulfuron	0.39	1.21	71(14)	85(5)	98(6)
80	Fludioxonil	0.39	1.21	111(8)	114(1)	100(2)
81	Flufenoxuron	0.05	0.16	97(6)	107(3)	107(4)
82	Fluopicolide	0.26	0.81	111(7)	108(5)	102(4)
83	Fluquinconazole	0.39	1.21	75(13)	90(8)	106(5)
84	Flusilazole	0.39	1.21	110(1)	109(5)	108(1)
85	Flutolanil	0.13	0.40	111(7)	90(10)	108(5)
86	Fluxapyroxad	0.39	1.21	92(8)	99(9)	109(2)
87	Forchlorfenuron	0.13	0.40	81(3)	88(2)	98(3)
88	Fosthiazate	0.13	0.40	85(10)	108(12)	105(6)
89	Furathiocarb	0.39	1.21	117(8)	113(4)	109(3)

90	Gibberellic acid	5.0	15.0	149(13)	121(8)	81(5)
91	Halosulfuron-methyl	0.39	1.21	99(1)	83(2)	97(4)
92	Haloxyfop	0.39	1.21	91(10)	82(9)	94(4)
93	Hexaconazole	0.39	1.21	118(8)	116(5)	112(3)
94	Hexaflumuron	0.39	1.21	114(9)	113(11)	102(3)
95	Hexazinone	0.26	0.81	119(14)	83(10)	100(5)
96	Hexythiazox	0.39	1.21	116(8)	91(7)	101(8)
97	Imazalil	0.13	0.40	79(12)	87(7)	105(1)
98	Imazosulfuron	0.26	0.81	82(12)	97(10)	100(4)
99	Imicyafos	0.39	1.21	91(6)	115(7)	113(7)
100	Imidacloprid	0.05	0.16	110(7)	107(4)	100(2)
101	Inabenfide	0.26	0.81	112(6)	89(5)	101(7)
102	Iprobenfos	0.39	1.21	100(7)	113(13)	109(6)
103	Iprovalicarb	0.26	0.81	83(7)	100(10)	103(6)
104	Isoprocarb	0.26	0.81	86(1)	89(4)	108(3)
105	Isoprothiolane	0.39	1.21	91(12)	116(10)	108(4)
106	Isopyrazam	0.39	1.21	114(2)	119(2)	116(4)
107	Kresoxim-methyl	0.39	1.21	91(6)	95(7)	110(2)
108	Linuron	0.05	0.16	86(11)	95(6)	111(4)
109	Lufenuron	0.39	1.21	116(10)	96(2)	108(7)
110	Malathion	0.39	1.21	88(5)	108(9)	102(2)
111	Mandipropamid	0.39	1.21	116(11)	95(11)	92(8)
112	Mefenacet	0.39	1.21	91(11)	112(7)	103(4)
113	Mepanipyrim	0.39	1.21	75(5)	106(2)	97(4)
114	Mepiquate chloride	0.39	1.21	119(8)	99(3)	100(5)
115	Metalaxyl	0.05	0.16	86(7)	88(3)	106(8)
116	Metamifop	0.26	0.81	112(4)	111(8)	113(7)
117	Metazosulfuron	0.39	1.21	103(6)	100(3)	84(2)
118	Metconazole	0.26	0.81	95(12)	104(2)	99(2)
119	Methabenzthiazuron	0.26	0.81	109(11)	76(5)	92(4)
120	Methiocarb	0.39	1.21	74(8)	107(5)	116(6)
121	Methomyl	0.39	1.21	82(9)	114(3)	92(1)
122	Methoxyfenozide	0.39	1.21	83(6)	95(6)	105(5)
123	Metobromuron	0.05	0.16	115(14)	95(7)	116(6)
124	Metolcarb	0.39	1.21	90(11)	115(4)	111(5)
125	Metrafenone	0.39	1.21	97(6)	110(1)	111(2)
126	Mevinphos	0.39	1.21	90(11)	94(4)	95(2)
127	Milbemectin A3	0.39	1.21	114(10)	81(7)	104(7)
128	Milbemectin A4	0.39	1.21	104(2)	102(8)	115(5)
129	Molinate	0.39	1.21	90(8)	98(7)	108(5)
130	Monocrotophos	0.39	1.21	79(2)	97(3)	79(1)
131	Myclobutanil	0.13	0.40	92(11)	104(3)	119(4)
132	Napropamide	0.05	0.16	95(3)	99(3)	107(3)
133	Nicosulfuron	0.05	0.16	108(6)	100(9)	103(5)
134	Novaluron	0.26	0.81	108(12)	116(8)	119(5)
135	Nuarimol	0.39	1.21	101(10)	91(8)	102(10)
136	Ofurace	0.39	1.21	100(9)	119(5)	103(6)
137	Omethoate	0.39	1.21	112(13)	116(11)	96(6)
138	Oxadiazon	0.39	1.21	111(9)	99(6)	116(6)
139	Oxamyl	0.39	1.21	114(13)	114(4)	93(2)
140	Oxaziclomefone	0.39	1.21	114(3)	113(3)	103(3)

141	Paclobutrazol	0.39	1.21	93(6)	107(5)	113(5)
142	Penconazole	0.39	1.21	118(12)	117(4)	110(3)
143	Pencycuron	0.39	1.21	100(4)	107(6)	114(1)
144	Penoxsulam	0.39	1.21	71(6)	114(5)	106(1)
145	Pentoxazone	0.39	1.21	86(13)	103(5)	109(6)
146	Phenthoate	0.13	0.40	106(14)	118(10)	113(6)
147	Phosphamidon	0.39	1.21	83(6)	88(7)	108(4)
148	Phoxim	0.39	1.21	98(2)	115(5)	103(3)
149	Piperophos	0.39	1.21	104(13)	107(3)	104(3)
150	Pirimicarb	0.39	1.21	118(10)	89(4)	93(4)
151	Pirimiphos-methyl	0.39	1.21	114(12)	119(6)	113(8)
152	Probenazole	0.39	1.21	99(3)	114(3)	104(3)
153	Profenofos	0.39	1.21	119(11)	118(5)	107(8)
154	Propamocarb	0.39	1.21	81(5)	87(3)	90(4)
155	Propanil	0.39	1.21	99(1)	99(1)	117(1)
156	Propaquizafop	0.39	1.21	135(9)	119(10)	112(4)
157	Propoxur	0.39	1.21	80(11)	116(7)	114(3)
158	Pyraclofos	0.39	1.21	105(8)	113(9)	114(10)
159	Pyraclostrobin	0.39	1.21	84(8)	112(1)	101(6)
160	Pyrazolate	0.39	1.21	112(7)	108(5)	106(1)
161	Pyrazophos	0.39	1.21	93(2)	113(7)	100(3)
162	Pyribenzoxim	0.39	1.21	106(2)	107(6)	118(2)
163	Pyributicarb	0.39	1.21	92(7)	108(6)	100(7)
164	Pyridaben	0.13	0.40	111(9)	103(3)	104(7)
165	Pyridaphenthion	0.26	0.81	118(1)	101(5)	101(4)
166	Pyrifluquinazon	0.39	1.21	101(4)	95(8)	105(4)
167	Pyriftalid	0.39	1.21	85(11)	100(8)	107(3)
168	Pyrimethanil	0.13	0.40	73(12)	83(3)	102(5)
169	Pyrimidifen	0.13	0.40	118(3)	101(3)	99(7)
170	Pyriminobac-methyl(E)	0.39	1.21	95(1)	100(4)	103(5)
171	Pyriminobac-methyl(Z)	0.39	1.21	93(10)	92(3)	105(6)
172	Pyrimisulfan	0.39	1.21	87(12)	90(10)	104(7)
173	Pyriproxyfen	0.39	1.21	117(3)	103(3)	109(2)
174	Pyroquilon	0.39	1.21	135(4)	119(6)	110(4)
175	Quinalphos	0.39	1.21	107(13)	119(12)	104(5)
176	Quinmerac	0.39	1.21	88(1)	83(2)	91(3)
177	Quinoclamine	0.39	1.21	116(5)	90(2)	102(2)
178	Quizalofop-ethyl	0.39	1.21	119(6)	112(8)	118(6)
179	Saflufenacil	0.39	1.21	106(5)	112(4)	108(3)
180	Sethoxydim	0.39	1.21	101(12)	102(5)	101(3)
181	Spinetoram_J	0.39	1.21	115(6)	111(4)	100(4)
182	Spinetoram_L	0.39	1.21	104(13)	87(8)	97(5)
183	Spirodiclofen	0.39	1.21	116(7)	117(5)	118(6)
184	Spirotetramat	0.39	1.21	82(11)	112(8)	113(6)
185	Sulfoxaflor	0.39	1.21	83(6)	101(9)	101(3)
186	Tebuconazole	0.13	0.40	119(12)	106(11)	91(5)
187	Tebufenozide	0.13	0.40	97(5)	105(5)	109(6)
188	Tebufenpyrad	0.13	0.40	118(12)	91(10)	101(7)
189	Teflubenzuron	0.39	1.21	109(11)	110(6)	109(1)
190	Terbuthylazine	0.39	1.21	81(1)	85(4)	105(5)
191	Tetraconazole	0.13	0.40	91(9)	83(5)	106(2)

192	Thenylchor	0.39	1.21	92(10)	102(2)	105(5)
193	Thiabendazole	0.39	1.21	81(1)	99(5)	81(1)
194	Thiacloprid	0.13	0.40	84(6)	100(2)	99(2)
195	Thiamethoxam	0.04	0.12	80(6)	102(4)	93(2)
196	Thiazopyr	0.39	1.21	112(4)	118(3)	106(3)
197	Thidiazuron	0.39	1.21	117(8)	118(10)	88(7)
198	Thifensulfuron-methyl	0.39	1.21	105(5)	104(4)	88(6)
199	Thiobencarb	0.39	1.21	113(13)	119(5)	108(2)
200	Thiodicarb	0.39	1.21	77(8)	97(7)	108(5)
201	Tiadinil	0.39	1.21	102(11)	106(6)	107(6)
202	Triadimefon	0.39	1.21	90(12)	105(8)	108(9)
203	Triazophos	0.13	0.40	96(8)	104(3)	102(5)
204	Tricyclazole	0.39	1.21	93(6)	105(6)	99(2)
205	Trifloxystrobin	0.05	0.16	119(1)	117(2)	102(7)
206	Triflumizole	0.26	0.81	113(6)	115(6)	108(4)
207	Triflumuron	0.13	0.40	116(6)	114(5)	106(1)

LOD: limit of detection, LOQ: limit of quantification, RSD: relative standard deviation.
